# Is the learning curve of the urology resident for conventional radical prostatectomy similar to that of staff initiating robot-assisted radical prostatectomy?

**DOI:** 10.1590/S1677-5538.IBJU.2024.9909

**Published:** 2024-03-10

**Authors:** Rodrigo da Silva Pires, Cláudio William Alves Pereira, Luciano Alves Favorito

**Affiliations:** 1 Hospital Naval Marcílio Dias Rio de Janeiro RJ Brasil Hospital Naval Marcílio Dias (HNMD), Rio de Janeiro, RJ, Brasil; 2 Universidade do Estado do Rio de Janeiro Unidade de Pesquisa Urogenital Rio de Janeiro RJ Brasil Unidade de Pesquisa Urogenital, Universidade - Universidade do Estado do Rio de Janeiro – UERJ, Rio de Janeiro, RJ, Brasil

**Keywords:** Prostatic Neoplasms, Robotic Surgical Procedures, Urology

## Abstract

**Introduction:**

The superiority of the functional results of robot-assisted radical prostatectomyis still controversial. Despite this, it is known that minimally invasive surgery obtains better results when analyzing blood loss, blood transfusion and length of stay, for example. Several studies have analyzed the impact of the resident physician's involvement on the results of urological surgeries. The simple learning curve for robot-assisted radical prostate surgery is estimated to be around 10 to 12 cases. Learning curve data for robotic surgeons is heterogeneous, making it difficult to analyze. Rare studies compare the results of a radical prostatectomy of an inexperienced surgeon starting his training in open surgery, with the results of the same surgeon, a few years later, starting training in robotic surgery.

**Objective:**

to analyze the results of open radical prostatectomy surgeries (ORP) performed by urology residents, comparing them to the results of robot-assisted radical prostatectomy (RARP), performed by these same surgeons, after completing their training in urology.

**Materials and methods:**

a retrospective analysis of the cases of only 3 surgeons was performed. 50 patients underwent ORP (group A). The surgeons who operated on the ORP patients were in the 3rd and final year of the urology residency program and beginners in ORP surgery, but with at least 4 years of experience in open surgery. The same surgeons, already trained urologists, began their training in robotic surgery and performed 56 RARP surgeries (group B). For the comparative analysis, data were collected on age, number of lymph nodes removed, surgery time, hospitalization time, drain volume, drain permanence time, indwelling bladdercateter (IBC) permanence time, positive surgical margin, biochemical recurrence, risk classification (ISUP), intra and postoperative complications, urinary incontinence (UI) and erectile dysfunction (ED). The console used was the Da Vinci Si, from Intuitive^®^.

For statistical analysis, the Shapiro-Wilk test verified that the data did not follow normality, the Levene test guaranteed homogeneity, and the Mann-Whitney test performed the comparative analysis of the quantitative data. For the analysis of qualitative data, the Chi-square test was used for nominal variables and the Mann-Whitney U test for ordinal variables. Additionally, the Friedman test analyzed whether there was an improvement in the perception of UI or ED over the months, for each group individually (without comparing them), and the post-hoc Durbin-Conover test, for the results with statistically significant difference. We used a p-value < 0.05, and the Jamovi^®^ program (Version 2.0).

**Results:**

there was no statistically significant difference between the groups for age, number of lymph nodes removed, positive surgical margin, biochemical recurrence, risk classification and urinary incontinence. Additionally, we observed that the surgical time was longer in group B. On the other hand, the length of stay, drain volume, drain time, IBC time, complication rate and levels of erectile dysfunction in the third and sixth months were higher in group A, when compared to group B. We also observed that there was no evolutionary improvement in ED over the months in both groups, and that there was a perception of improvement in UI from the 1st to the 3rd month in group A, and from the 1st to the 6th month, and from the 3rd to the 12th month, in group B.

**Conclusion:**

the learning curve of RARP is equivalent to the curve of ORP. In general, the results for the robotic group were better, however, the functional results were similar between the groups, with a slight tendency of advantage for the robotic arm.

## INTRODUCTION

Much is discussed about the superiority of the oncological results of RARP compared to ORP, but the results are still insufficient to confirm. Likewise, the functional results are also controversial since the first randomized clinical trial comparing the two techniques, performed by Yaxley, et al, in 2016 (1). Despite that, it is known that minimally invasive surgery obtains better results when analyzing blood loss, blood transfusion and length of stay, for example. On the other hand, the surgery time is usually longer in these procedures when compared to conventional procedures (2-8).

Several studies analyzing the impact of the resident physician's involvement on the results of minimally invasive urological surgeries have shown that the operative time is longer in these procedures. However, the rates of complications, reoperation and length of hospital stay are no worse than surgeries not involving residents (9-14).

Recently, Johnson (15) estimated that the simple learning curve for RARP surgery is around 10 to 12 cases. Recently, a systematic review demonstrated that the learning curve, based on operative time, was identified as being from 10 to 250 cases for RARP (16). To make this small number possible, medicine has incorporated the technology of simulators, which has helped surgeons in their learning curve, contributing to patient safety (17-19).

Learning curve data for robotic surgeons is heterogeneous, making it difficult to analyze. The effect of open surgery experience on surgeons who are learning robotic surgery is not yet clear, but these surgeons appear to have favorable oncological and functional results compared to those entering robotic surgery with no open surgery experience (20-22).

Rare studies compare the results of a radical prostatectomy of an inexperienced surgeon starting his training in open surgery, with the results of the same surgeon, a few years later, starting training in robotic surgery. The study by Phillipou et al (23), was able to perform this comparison for a single surgeon. To the best of our knowledge, we did not detect studies that performed this analysis with a group of surgeons, comparing the learning curve of the same surgeons in open and robotic surgery.

The aim of this study is to analyze the results of the first ORP performed by urology residents, comparing them to the results of the first RARP surgeries performed by these same surgeons, after completing their training in urology.

## MATERIAL AND METHODS

This study was carried out in accordance with the ethical standards of the institutional hospital human experimentation committee (IRB: 4.910.918, CAAE: 49880321.4.0000.5256). After a retrospective analysis of the electronic and physical medical records of 106 patients, 50 of whom underwent open retropubic radical prostatectomy (ORP) between 2011 and 2017, were allocated in the group A. The other 56 patients underwent robot-assisted radical prostatectomy (RARP) between 2016 and 2021 and were allocated in group B.

Only the patients of three surgeons were analyzed. The surgeons who operated on ORP patients, respectively, between 2011 and 2016 were in the 3rd and final year of the urology residency program and beginners in ORP surgery, but with experience in open surgery of at least 4 years (first 23 cases of surgeon 1, first 15 cases of surgeon 2, and first 12 cases of surgeon 3). The same surgeons, respectively between 2016 and 2021, already trained urologists, began their training in robotic surgery (first 25 cases of surgeon 1, first 16 cases of surgeon 2, and first 12 cases of surgeon 3).

It is worth noting that the proctors of robotic surgery were surgeons with experience in more than 50 robotic surgeries and that the first assistants in open surgeries were staff with experience in more than 50 open PTRs. In addition, the other assistants from both groups were, at random, resident physicians in urology.

Pre, intra and postoperative data were analyzed, as follows:

### Quantitative data:

- Age: in years on the day of surgery.
- Number of lymph nodes removed: total lymph nodes removed (impaired by neoplasm and not impaired) in obturator lymphadenectomy.
- Surgery time (Surg T): calculated in minutes between the beginning and the end of the surgery, noted in the nursing record. For both surgeries, the time considered was from the beginning of the skin incision to complete skin synthesis. That is, in robotic surgeries, robot docking times and access to the cavity were also included.
- Length of stay (L Sty): calculated by subtracting the date of discharge from the date of surgery.
- Drain Volume (Drain V): considered the volume measured on the 1st postoperative day. The zero value was considered in patients left without a drain.
- Drain permanence time (Drain T): was measured in days.
- Indwelling Bladder Catheter permanence time (IBC T): calculated in days.

### Qualitative Data:

- Positive surgical margin (PSM): defined as the presence of tumor tissue on the surface of the stained and ink piece, on the urethral, vesical or circumferential margins.
- Biochemical recurrence (BR): defined as PSA greater than or equal to 0.2, in the first or third month after surgery (24).
- Risk Classification: the histopathological degree of ISUP was considered (25, 26).
- Intra or postoperative complications: during surgery or up to 1 year after surgery, they were classified according to the Clavien-Dindo classification (27).
- Urinary incontinence (UI): considered continent patient - 0 (up to one pad a day), mild incontinent - 1 (two pads a day) or severe incontinent - 2 (three or more pads a day), through an interview, in 1, 3, 6 and 12 postoperative months.
- Erectile dysfunction (ED): defined as absent - 0, partial - 1 or severe - 2, after interview with the patient, in the 3, 6 and 12 postoperative months.

The console used was the Da Vinci Si, from Intuitive^®^, using scissors on robotic arm 1, Maryland, on 2, and grasping forceps, on 3. Two needle holders were used to perform the anastomosis. Stratafix barbed thread was used in robotic surgeries to create the anastomosis and PDS to close the bladder neck, when necessary. The technique used was the classical one by the anterior route (28). In conventional surgeries, common open surgery materials were used, such as autostatic retractors, curved scissors, curved babcock forceps and needle holder. Anastomoses were performed with separate PDS or Vicryl stitches.

### Statistical Analysis

The statistical study was carried out using the Jamovi^®^ program (Version 2.0). First, we performed a descriptive analysis of all quantitative data, summarized in Table-1.

**Table 1:** Quantitative data on the number of participants, minimum, maximum, median, mean, standard deviation and p value.

	Group	Minimum	Maximum	Median	Mean	Standard deviation	p value
**Age (years)**	A	43	75	65	62.0	8.44	0.091
	B	39	76	60	59.6	8.19	
**Lymph nodes**	A	0	12	0	2.64	3.59	0.986
	B	0	15	1	2.21	3.44	
**Surg T (min)**	A	105	330	166	172	46.8	< 0.01
	B	150	345	245	246	44.1	
**L Sty (days)**	A	2	18	4	5.40	4.38	< 0.001
	B	1	23	2	3.09	3.79	
**Drain V (mL)**	A	0	1000	142,5	192	181	< 0.001
	B	0	700	30	95.2	161	
**Drain T (days)**	A	1	16	2	3.80	3.70	< 0.001
	B	0	14	1	2.00	2.49	
**IBC T (days)**	A	13	30	16	16.1	3.15	< 0.001
	B	6	23	8	8.96	3.14	

To establish the normality of the data, we performed the Shapiro-Wilk test and verified that all quantitative data did not follow a normal distribution (p < 0.05). As we can verify by Levene's test, we obtained homogeneity of all quantitative data between the two groups, with p > 0.05. Therefore, we performed the Mann-Whitney U test for the comparative analysis of all non-parametric quantitative data.

## RESULTS

Therefore, we performed the Mann-Whitney U test for the comparative analysis of all non-parametric quantitative data.

Through this statistical analysis, we can see that there was no statistically significant difference in the comparison between the ages of groups A (age: 43 to 75 years; mean = 62.02; SD = 8.44) and B (age: 39 to 76 years; mean = 59.58; SD = 8.19) with p = 0.091.

Likewise, we noticed that there was no statistically significant difference in the comparison between the number of lymph nodes removed in surgeries with lymphadenectomy in groups A (lymph nodes: 0 to 12; mean = 2.64; SD = 3.59) and B (lymph nodes: 0 to 15; mean = 2.21; SD = 3.44), with p = 0.986.

**Figure 1:**
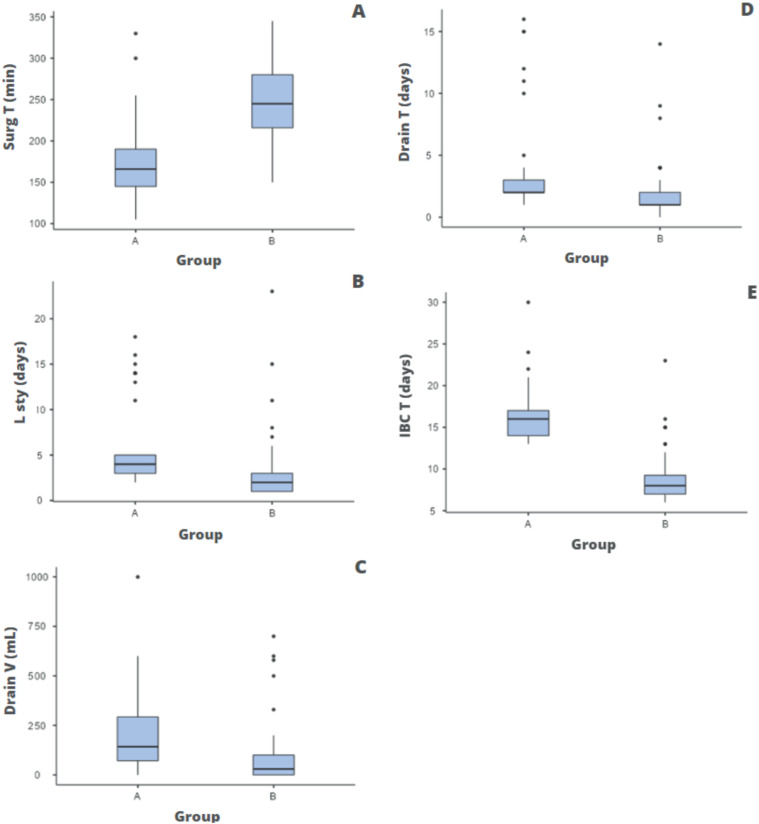
The figure shows the statistical comparison with the Mann-Whitney U test, between group A (open retropubic radical prostatectomy) and group B (robot-assisted radical prostatectomy), of the following parameters: A) Total surgery time (p < 0.01); B) Length of hospital stay (p < 0.001); C) Drain volume on the first postoperative day (p < 0.001); D) Drain permanence time (p < 0.001) and E) Indwelling bladder catheter (IBC) permanence time (p < 0.001).

On the other hand, we observed that all other quantitative data had a statistically significant difference between the two groups (Figure-1).

The analysis of surgery time (Surg T) indicated that group A (Surg T: 105 to 330 min; mean = 172; SD 46.8) had a shorter surgery time than group B (Surg T: 150 to 345 min; mean = 246; SD 44.1), with p value < 0.01.

Inversely to what was found for surgery time, data on length of stay (L sty), drain volume on the first postoperative day (drain V), drain time (drain T) and indwelling bladder catheter permanence time (IBC T), were higher in group A when compared to group B, with the following results:

L sty A (2 to 18 days; mean = 5.40; SD 4.38) and L sty B (1 to 23; mean = 3.09; SD = 181), p < 0.001;
Drain V A (0 to 1000 mL; mean = 192; SD 210) and Drain V B (0 to 700 mL; mean = 95.2; SD 161), p < 0.001;
Drain T A (1 to 16 days; mean = 3.80; SD 3.70) and Drain T B (0 to 14 days; mean = 2.0; SD 2.49), p <0.001;
IBC T A (13 to 30 days; mean = 16.1; SD 3.15) and IUC T B (6 to 23 days; mean = 8.96; SD 3.14), p < 0.001.

**Caption:** statistical comparison with the Mann-Whitney U test, between groups A and B, of the following parameters: A – total surgery time (p < 0.01); B – length of hospital stay (p < 0.001); C – drain volume on the first postoperative day (p < 0.001); D – drain permanence time (p < 0.001); and E – IBC permanence time (p < 0.001).

For the analysis of qualitative data, the following steps were taken.

The Chi-Square test was used for the association of nominal qualitative variables, Positive Surgical Margin (PSM) and Biochemical Recurrence (BR), between groups A and B.

From this analysis, we can see that there was no statistically significant difference between the groups for PSM (group A: 49% of PSM; and group B: 47% of PSM), and for BR (group A: 27% of BR; and group B: 17% of BR), as the p value was > 0.05.

Ordinal qualitative data (ISUP and Clavien-Dindo ratings, as well as Urinary Incontinence at months 1, 3, 6, and 12 and Erectile Dysfunction at months 3, 6, and 12 after surgery) were analyzed using the Mann-Dindo test. Whitney U. The respective descriptive analyzes were compiled in Table-2.

**Table 2:** Descriptive analysis of ISUP, Clavien-Dindo, Urinary incontinence and Erectile dysfunction.

	N		25th percentile		50th percentile		75th percentile	
**Group**	A	B	A	B	A	B	A	B
**ISUP**	50	51	1.00	1.00	2.00	2.00	3.00	3.00
**Clavien-Dindo**	50	49	0.00	0.00	1.00	0.00	3.00	1.00
**UI 1 month**	30	37	0.00	0.00	1.50	1.00	2.00	1.00
**UI 3 months**	29	36	0.00	0.00	0.00	0.00	1.00	1.00
**UI 6 months**	32	33	0.00	0.00	0.00	0.00	1.00	1.00
**UI 12 months**	35	30	0.00	0.00	0.00	0.00	1.00	0.00
**ED 3 months**	27	38	2.00	1.00	2.00	2.00	2.00	2.00
**ED 6 months**	29	35	2.00	1.00	2.00	1.00	2.00	2.00
**ED 12 months**	34	28	1.00	1.00	2.00	2.00	2.00	2.00

It is observed that there was no statistically significant difference between groups A and B, for the risk classification, according to ISUP (p > 0.05).

However, we found that for group A, there was a greater number of complications when compared to group B, according to the Clavien-Dindo (CD) classification (p < 0.05). Despite this, we observed one death associated with group B (CD grade 5), and both groups had CD grade 4 complications (1 case of acute renal failure in group A and 1 case of hypovolemic shock in group B). We also found 1 case of conversion to conventional surgery.

We observed that there was no statistically significant difference between groups A and B, in terms of urinary incontinence, in the 1st, 3rd, 6th and 12th postoperative months (p > 0.05).

From the analysis of the percentiles, we can observe that group B had less erectile dysfunction in the 3rd and 6th postoperative months, and that this difference was statistically significant (p < 0.05). We also found that there was no statistically significant difference at the 12th month between the two groups for the erectile dysfunction item (p > 0.05).

Additionally, we performed the Friedman test, to analyze whether there was an improvement in the perception of UI or ED over the months, for each group individually (without comparing them), and the post-hoc Durbin-Conover test, for the results with statistically significant difference.

In view of this, the improvement in the UI of group A (A UI) occurred (p = 0.004), as we observed a decrease in the averages over the months. However, this improvement was only statistically significant from the 1st month to the 3rd month (p = 0.017). After the 3rd month, no statistically significant improvement was observed.

In the same way, the improvement in the UI of group B (B UI) occurred (p < 0.01), as we observed a decrease in the averages over the months. However, this improvement was only statistically significant from the 1st month to the 6th month (p = 0.006) and from the 3rd month to the 12th month.

When analyzing the evolution of ED in group A over the months, we observed that there was no evolutionary improvement in this group (p = 0.135).

Finally, when analyzing the evolution of ED in group B over the months, we observed that there was no improvement in this group either (p = 0.513).

## DISCUSSION

One of our concerns at the beginning of the study was that, possibly, there was a bias in the selection of patients for the robotic surgery learning group (group B), as it is a surgical technique established with a relatively short time, in the service of urology in question. Therefore, it was estimated that the best cases were unconsciously indicated for this technique. However, this question did not prove to be appropriate, as the statistics of our study showed a homogeneity of patients in groups A and B. Thus, we found no difference in both groups, comparing data on age, ISUP and lymph nodes parameters, which are predictors of worse prognosis or more advanced disease (29-34).

From the results, we can observe that, in general, we obtained better outcomes for group B. This was intuitively expected and is in line with the meta-analysis by Cao, et al (2), among others, since data such as length of stay, volume and postoperative drain time, and indwelling bladder catheter permanence time, are factors that generally are better for minimally invasive surgeries (3-7, 35).

Bleeding volume was not compiled due to the difficulty in establishing it accurately in retrospective studies, and especially when comparing robotic surgeries (in which the estimate is more accurate) with open surgeries (in which surgical compresses are used in addition to aspiration). For these reasons, we considered the drainage volume on the first postoperative day, reducing this bias that would benefit open surgery.

The surgery time was, on average, longer in group B than in group A. This data is consistent with the world literature, since the time of access to the cavity and docking of the robot was also taken into account (3-7, 35).

Biochemical recurrence (BR) after primary treatment with radical prostatectomy occurs in about 20 to 40% of cases after 10 years of treatment. This recurrence directly influences the indication of adjuvant treatment or rescue therapy (24). In our study, we observed the first and second PSA measured 1 month and 3 months after surgery, respectively. We considered RB to be PSA greater than 0.2 in one or another measure and, in this analysis, we did not observe any difference between the groups, as Bravi, et al (34) also concluded in their study. Thus, we can think that the indications for adjuvant treatments in both groups were similar (36). Furthermore, we reinforce the findings of the elegant and recent study by Bravi, et al (37), which shows that, contrary to what occurs in ORP and laparoscopic radical prostatectomies (LRP), surgeons who perform RARP achieve an appropriate control of prostate cancer, since the beginning of their training.

The analysis of perioperative and postoperative complications verified up to one year after surgery also obtained a better result for group B, and these results are in agreement with the 2021 study by Wu, et al (38), which concluded that RARP is associated with lower rates of acute and chronic complications when compared to ORP and LRP. It also concerns the study by Matulewicz, et al (7), which shows that there was no increase in the rate of complications in surgeries operated and/or assisted by resident surgeons. Despite this, the only fatal complication (CD grade 5) reported was due to acute complications such as urethrovesical fistula and acute renal failure, which culminated in pulmonary thromboembolism in one of the patients in group B (35).

Recently, Baunack, et al (39) also compared learning groups of RARP and ORP. In it, the authors identified lower mean age and oncological risk for patients operated on with the robotic technique rather than the open technique. Differently, in our study, we observed homogeneous data on age and oncological risk for both groups, which reduces the risks of a selection bias, with a possible impact on the results.

Furthermore, Baunack, et al (39), identified that surgeons inexperienced in robotics obtained a higher percentage of PSM and lower yield of dissected lymph nodes. In our study, there was no statistically significant difference in PSM and dissected lymph nodes when comparing the two groups, which gives more credit to robotic surgery Furthermore, Baunack, et al (39), identified that surgeons inexperienced in robotics obtained a higher percentage of PSM and lower yield of dissected lymph nodes. In our study, there was no statistically significant difference in PSM and dissected lymph nodes when comparing the two groups, which gives more credit to robotic surgery, and is in agreement with a recent study by Bravi et al (36), which also concluded that surgeons performing RARP achieve adequate cancer control in the early phase of their career. In this study, they also suggest that, differently from what occurs in the ORP and the LRP, the good results regarding the surgical margins in the RARP may be related to the training in previous simulation.

It should be noted that the total number of lymph nodes is what matters in the analysis of surgeons' experiences, since the percentage of affected lymph nodes is much more related to the degree of the disease and not to the surgeon's expertise. In this sense, there are still no studies for the learning curve in lymphadenectomy (39), in open surgery, with only two studies for RARP (40, 41), as far as we know.

Widely discussed in other studies, the issue of ED showed, in our study, an improvement in favor of group B in the third and sixth months after surgery. However, at twelve months, there was no difference between the two groups, as observed in much of the literature (23, 42).

It is worth mentioning that we did not consider patients who already had some degree of ED before surgery. However, given the age and degree of the disease, we assumed that the groups were similar. We also considered that patients who required androgen deprivation therapy (ADT) in the postoperative period were considered to have severe ED or absent data. In addition, patients who did not have sexual intercourse in the postoperative period had this data absent, and the positive response to the drug-induced induction test with intracavernous alprostadil, were considered to have partial ED. Despite these considerations, as we obtained homogeneity of age and degree of disease in both groups, we assume that both were similar.

There was no difference in the comparison of UI between the groups, which agrees with what is seen in the literature (23, 43). Despite this, we did not observe a difference in continence in the first month, which is usually an advantage for the robotic group (44). Even so, the IBC permanence time was, on average, almost half in group B, which adds greater comfort to patients in the postoperative period of this group.

In addition, we observed a perception of improvement in UI from the first to the third month in group A, and from the first to the sixth and from the third to the twelfth, in group B. On the other hand, there was no improvement in the perception of ED throughout of months in both groups. In this sense, we did not find any factor that could explain this perception of improvement in the groups, but we understand that this perception is subjective and, we assume that it may be related to the degree of expectation of patients when performing an open or robotic surgery. However, there is no concrete data, as this expectation was not evaluated in our study (45).

It is worth mentioning the limitations of the study. As this is a retrospective study, it is difficult to find all the data in the medical records. There was no application of validated questionnaires for ED and UI.

We must also take into account that in open surgery, the first aid was performed by a more experienced surgeon, who provides the entire surgical field. In robotic surgery, the first assistant, in our case, was a resident also on an assistant learning curve (10). Despite this, the proctor has the authority to "steal" parts of the surgery on the second console, as the first aid also does occasionally during the open surgery.

In addition, it is worth remembering that robotic surgeons already had experience in open surgery and some experience in laparoscopic surgery, which could influence the results of robot-assisted surgery.

## CONCLUSIONS

The learning curve of RARP is equivalent, safe and as difficult as the learning curve of ORP. As expected, the results for the robotic group were better when compared to the open surgery, as it is a minimally invasive surgery. However, the functional results were similar between the groups, with a slight tendency of advantage for the robotic arm.
